# Data-Driven Network Modeling as a Framework to Evaluate the Transmission of Piscine Myocarditis Virus (PMCV) in the Irish Farmed Atlantic Salmon Population and the Impact of Different Mitigation Measures

**DOI:** 10.3389/fvets.2020.00385

**Published:** 2020-07-16

**Authors:** Tadaishi Yatabe, Beatriz Martínez-López, José Manuel Díaz-Cao, Fiona Geoghegan, Neil M. Ruane, Teresa Morrissey, Catherine McManus, Ashley E. Hill, Simon J. More

**Affiliations:** ^1^Department of Medicine and Epidemiology, Center for Animal Disease Modeling and Surveillance (CADMS), School of Veterinary Medicine, University of California, Davis, Davis, CA, United States; ^2^Fish Health Unit, Marine Institute, Galway, Ireland; ^3^Mowi Ireland, Letterkenny, Ireland; ^4^California Animal Health and Food Safety Laboratories (CAHFS), Department of Medicine and Epidemiology, School of Veterinary Medicine, University of California, Davis, Davis, CA, United States; ^5^Centre for Veterinary Epidemiology and Risk Analysis (CVERA), UCD School of Veterinary Medicine, University College Dublin, Dublin, Ireland

**Keywords:** Ireland, network modeling, piscine myocarditis virus (PMCV), farmed salmon, disease transmission

## Abstract

Cardiomyopathy syndrome (CMS) is a severe cardiac disease of Atlantic salmon caused by the piscine myocarditis virus (PMCV), which was first reported in Ireland in 2012. In this paper, we describe the use of data-driven network modeling as a framework to evaluate the transmission of PMCV in the Irish farmed Atlantic salmon population and the impact of different mitigation measures. Input data included live fish movement data from 2009 to 2017, population dynamics events and the spatial location of the farms. With these inputs, we fitted a network-based stochastic infection spread model. After assumed initial introduction of the agent in 2009, our results indicate that it took 5 years to reach a between-farm prevalence of 100% in late 2014, with older fish being most affected. Local spread accounted for only a small proportion of new infections, being more important for sustained infection in a given area. Spread via movement of subclinically infected fish was most important for explaining the observed countrywide spread of the agent. Of the targeted intervention strategies evaluated, the most effective were those that target those fish farms in Ireland that can be considered the most connected, based on the number of farm-to-farm linkages in a specific time period through outward fish movements. The application of these interventions in a proactive way (before the first reported outbreak of the disease in 2012), assuming an active testing of fish consignments to and from the top 8 ranked farms in terms of outward fish movement, would have yielded the most protection for the Irish salmon farming industry. Using this approach, the between-farm PMCV prevalence never exceeded 20% throughout the simulation time (as opposed to the simulated 100% when no interventions are applied). We argue that the Irish salmon farming industry would benefit from this approach in the future, as it would help in early detection and prevention of the spread of viral agents currently exotic to the country.

## Introduction

Aquaculture is an important contributor to the Irish economy, producing products to the value of €167 million in 2016, including €105 million from farmed Atlantic salmon (*Salmo salar* L.). The industry is particularly important along the western seaboard of Ireland. Most Irish salmon farming is certified organic (1, 2).

Salmon farming in Ireland is associated with an intricate network of fish movements within and between the different types of salmon farms. There are three different farm types, including broodstock, freshwater, and seawater farms. In earlier work (3), social network analysis was used in combination with spatial epidemiological methods to characterize the network structure of live farmed salmonid movements in Ireland. It was demonstrated that characteristics of the network of live salmonid fish movements in Ireland would facilitate infection spread processes. These included a power-law degree distribution [that is, “scale free”], short average path length and high clustering coefficients [that is, “small world”], with the presence of farms that could potentially act as super-spreaders or super-receivers of infection, with few intermediaries of fish movement between farms, where infectious agents could easily spread, provided no effective barriers are placed within these farms (3). A small proportion of sites play a central role in the trade of live fish in the country. Similarly, we demonstrated that highly central farms are more likely to have a number of different diseases affecting the farm during a year, diminishing the effectiveness of in-farm biosecurity measures (decreasing its economic return), and that this effect might be explained by an increased chance of new pathogens entering into the farm environment (4). This is a very important area of research in aquaculture, especially considering that the spread of infection via fish movement is considered one of the main routes of transmission (5, 6).

Mathematical models and computer simulations offer the potential to study the spread of infectious diseases and to critically evaluate different intervention strategies (7–9). Through access to real fish movement data, these models can be programmed to incorporate both the time-varying contact network and data-driven population demographics. However, there are considerable computational challenges when stochastic simulations are conducted using livestock data, both computationally, including the need for efficient algorithms, and also with model selection and parameter inference (10). An efficient modeling framework for event-based epidemiological simulations of infectious diseases has recently been developed (11, 12), including the use of a framework that integrates within-farm infection dynamics as continuous-time Markov chains and livestock data as scheduled events. This approach was recently used to model the spread of Verotoxigenic *Escherichia coli* O157:H7 (VTEC O157) in Swedish cattle (13, 14).

Cardiomyopathy syndrome (CMS) is a severe cardiac disease of Atlantic salmon. It was first reported in the mid-1980s in farmed salmon in Norway (15) and later detected in several other European countries, including the Faroe Islands (16), Scotland (17) and, in 2012, in Ireland (18). CMS generally presents as a chronic disease, leading to long-lasting, low-level mortality, although some individuals experience sudden death. At times, however, CMS can present as an acute, dramatic increase in mortality associated with stress (e.g., predators, other diseases, grading, treatments, and transportation) (19). A recent Norwegian study has identified risk factors for developing clinical CMS, including stocking time, time at sea, a previous outbreak of pancreatic disease (PD) or Heart and Skeletal Muscle Inflammation (HSMI), and hatchery of origin (20). The economic impact of CMS is particularly serious as it occurs late in the life cycle, primarily during the second year at sea, by which time the incurred expenditure is high. No effective preventive measures are known, and there is no treatment available (21). In 2009, CMS was identified as a transmissible disease (22, 23), and has been linked, in 2010 and 2011, to a virus resembling viruses of the Totiviridae family (24, 25). The discovery of this virus, piscine myocarditis virus (PMCV), has contributed to increased knowledge about the disease including the development of new diagnostic, research and monitoring tools (19). The agent is spread horizontally, between farms at sea, although there is some indication of a possible vertical transmission pathway (19, 26, 27). Recent Norwegian research has shown that PMCV is relatively widespread, including in geographic regions and fish groups without any evidence of CMS (20). The mechanisms leading to progression from PMCV infection to CMS are currently unclear (19).

CMS is present in Ireland. The first recorded outbreak of CMS occurred in 2012, associated with low-level mortalities (single figures per day per cage) over a period of 4–5 weeks followed by increased mortalities during bath treatment for sea lice (*Lepeophtheirus salmonis* Krøyer) (18). CMS is not a notifiable disease in Ireland, and there are no systematic records of its occurrence. Nonetheless, anecdotal information from field veterinarians and farmers suggest that CMS occurrence has steadily increased over the years. A retrospective study was recently conducted, using real-time RT-PCR with archived broodstock samples dating back to 2006, which suggests that PMCV may have been introduced into Ireland in two different waves, both from the southern part of the range for PMCV in Norway (28, 29). PMCV was found to be largely homogenous in Irish samples, with limited genetic diversity. Further, the majority of PMCV strains had been sequenced from fish that were not exhibiting any clinical signs of CMS, which suggests possible changes in agent virulent and/or the development of immunity in Irish farmed Atlantic salmon (29).

This paper describes the use of data-driven network modeling as a framework to evaluate the transmission of PMCV in the Irish farmed Atlantic salmon population and the impact of targeted intervention strategies. This approach can be used to inform control policies for PMCV in Ireland, as well as other infectious diseases in the future.

## Materials and Methods

### Farmed Salmon Production in Ireland

The characteristics of the Irish Atlantic salmon have been described elsewhere (3, 4), but briefly, in broodstock farms, eggs, and milt are obtained from sexually mature fish to produce fertilized eggs. In freshwater farms, fertilized eggs hatch, and fish are kept until smoltification, the stage where fish are ready to transition into the ocean (70–100 grams or 6–15 months of age). Some companies move the fish to net pens in freshwater lakes for the smoltification to occur there. After smoltification, fish are transported to seawater net pens, where they will grow until market size (four to five kilos at 18–24 months of age), with the possibility of being moved to other sea sites in between. Some of these fish are selected to become the broodstock for the next production cycle. These fish are transported from sea sites into freshwater broodstock facilities in late summer and early fall, to be stripped later in winter.

### Infection Spread Model

These methods are based on those described by Widgren et al. (13, 14).

**Figure 1:**
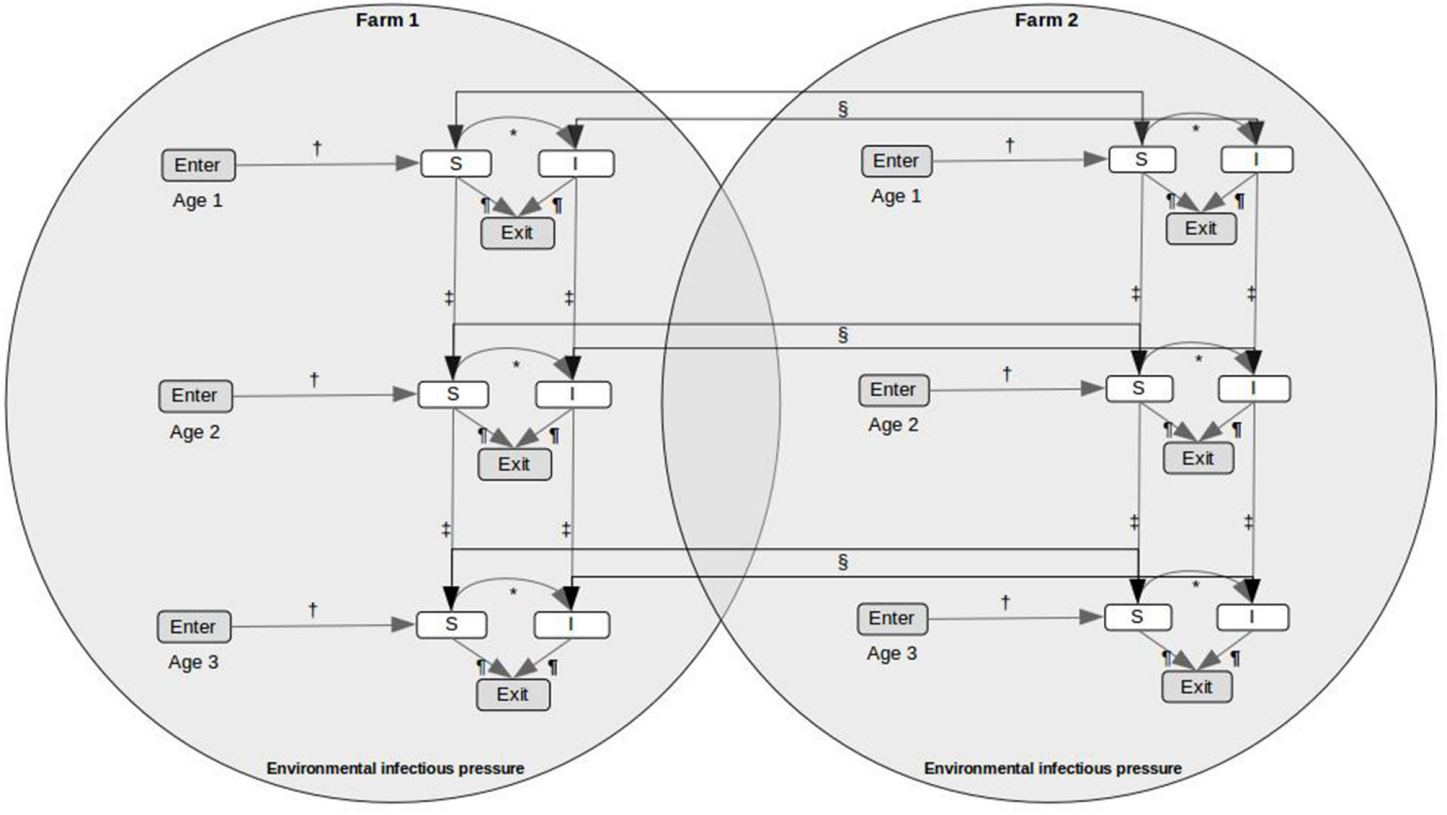
Conceptual model for PMCV spread in farmed Atlantic salmon, including indirect transmission via the environment and fish movements between holdings. The SI_E_ compartment model includes two disease states, susceptible (S) and infected (I), an environmental infectious pressure compartment (E), and the population is divided in three age categories. *State transitions between the infection states is modeled using a continuous-time discrete state Markov process with the Gillespie's direct method. State transitions due to scheduled events from fish movement data, including (†) enter (hatching on-farm, imports to the farm), (‡) aging, (§) movement between holdings, and (¶) exit (harvest or death, each on-farm), are represented.

A stochastic within-farm model, linked to other farms through fish movements and local spread, was used to model the dynamics of PMCV infection in each farm. We developed a SI_E_ compartment model with two disease states, susceptible (S) and infected (I), and an environmental compartment, E. It was assumed that infected fish do not recover (and therefore do not return to the susceptible state), and that susceptible fish can become infected through contact with PMCV present in the environment or via introduction of infected fish. To evaluate age-related differences in both the dynamics of PMCV infection within the host and in the likelihood of being moved (3), the two disease states, S and I, were further subdivided into three different age categories, indexed using *j*, including a. “egg-juveniles” from egg until 7 days prior to transfer to a marine farm, b. “smolt” from 7 days prior to the transfer to a marine farm to 180 days after the transfer, and c. “growth-repro” being more than 180 days in a marine farm. In those situations where this level of detail was not available for a particular fish group, all fish moving between freshwater farms were assumed to be egg-juveniles, and all moving between marine farms were assumed to be growth-repro. Therefore, *S*_*i, j*_, *I*_*i, j*_, and *E*_*i*_ represents the six disease compartments and the environmental compartment within each farm *i* (Figure 1). A continuous-time discrete-state Markov process with the Gillespie's direct method, as implemented by Bauer et al. (11), was used to model the state transitions between the susceptible and infected compartments within each farm (30, 31).

Within each farm *i*, the infectious pressure in the environmental compartment (environmental infectious pressure) φ_*i*_(*t*) was assumed to be time-dependent and uniformly distributed. Further, it was assumed that farm size was proportional to the number of fish in each farm. The environmental infectious pressure in each farm is represented as:

(1) $$\begin{array}{l} \frac{d\varphi_{i}\left(t\right)}{dt}=\frac{\alpha \sum_{j}I_{i,j}\left(t\right)}{N_{i}\left(t\right)}+\underset{k}{\sum }\frac{\varphi_{k}\left(t\right)N_{k}\left(t\right)-\varphi_{i}\left(t\right)N_{i}\left(t\right)}{N_{i}\left(t\right)}.\\ \;\frac{D}{d_{ik}}-\beta \left(t\right)\varphi_{i}\left(t\right) \end{array}$$

where the constant α is the average shedding rate per day per infected individual that contribute to the environmental infectious pressure, *N*_*i*_(*t*) is the total number of fish in farm *i* at time *t, D* is the rate of coupling (the spatial coupling parameter), *d*_*ik*_ is the distance between farms *i* and *k* (for all marine farms with *d*_*ik*_ ≤ the threshold distance), *D*/*d*_*ik*_ is the rate of local spread, and β(*t*) captures the rate per day of viral decay and therefore reduction in the environmental infectious pressure φ_*i*_(*t*).

Transition from the susceptible to infected state is related to the age-dependent, indirect transmission rate *v*_*j*_ and exposure to the environmental infectious pressure φ_*i*_(*t*):

(2) $$\begin{array}{l} S_{i,j}\overset{v_{j}\varphi_{i}}{\to }I_{i,j} \end{array}$$

Experimental studies have found PMCV as late as 33 weeks post-infection (wpi) (32), which suggests that the salmonid immune response may not be sufficient to eliminate the virus (33, 34). To maintain model parsimony, it was assumed that infected individuals do not return to the susceptible compartment.

### Parameter Estimation

Model parameters were estimated from a previous study, which had been conducted in 2016 and 2017 (28) to determine the prevalence of PMCV infection in Irish salmon farms by real-time RT-PCR (24). The sampling strategy was replicated to ascertain the status that could have been found if simulated farms had been sampled. In this study, sample collection was conducted on 22 farms (10 seawater, 8 freshwater, 2 broodstock, 1 research, and 1 ranching) from 30 May 2016 to 19 December 2017. A ranching farm is a freshwater broodstock farm that releases juvenile fish to the environment for conservation purposes. Some (7) farms were sampled more than once over the course of the study, with the median (range) samplings per farm in this group being 3.5 (2–9). A total of 1,201 fish were sampled (median 29 fish per farm, range 3–405) during the study. Samples consisted of heart tissue across all fish age classes (juvenile fish, smolts prior to sea transfer, and broodstock) and ova. In this study, PMCV was detected at a low level in most sites, with only one clinical case of CMS occurring during the study period. We simulated sampling at each time point by randomly sampling fish within each farm and age category, as in the observed data set, from the number of susceptible and infected individuals at the time for the sampling point in the simulated farms.

The aforementioned observational study also looked for PMCV in archived samples of Atlantic salmon broodstock from 2006 to 2016, seeking to determine whether the agent had been present in the country prior to the first case report in 2012 (18). For this, archived samples of broodstock Atlantic salmon were tested for each year from 2006 through 2016, using 60 archived pools (300 fish) per year. Samples are collected on an annual basis as part of the national disease surveillance programme, and consist of pooled (5 fish) organ homogenate (heart/kidney/spleen) supernatants which are then stored at −80°C. In these samples, PMCV was first detected from broodstock fish on a marine site in July 2009, with infection in 100% of the pools (10 in total). It was later detected in December of the same year in 100% of pools of broodstock fish at a second broodstock farm. These are subsequently referred to as the index cases, to be used as the starting point for simulation of the epidemic. The rationale behind setting these two farms as index cases is that they are the earliest detections (only 5 months apart), and do not seem to be epidemiologically connected.

There were 9 parameters in the SI_E_ compartment model (Table 1). Following the approach used by Widgren et al. (13), and for model parsimony, the shed rate α was fixed at 1.0 per day, thereby defining the unit of the environmental infectious pressure variable φ_*i*_(*t*). In the absence of more detailed data, it was assumed that the threshold for two seawater farms being connected by local spread was an euclidean distance of 10 km. It was further assumed that freshwater farms were not connected via local spread but only via fish movement. The parameters β, *v* and D (the spatial coupling parameter) were estimated by evaluating the agreement between observed and simulated fish status, the former based on the prevalence study results (28). The two time-series with observed and simulated fish statuses were defined as *Y*(*t*) and Y^*^(*t*, θ) where θ was the vector of model parameters in the simulation. With these values, a loss function was defined as the sum of the squared difference between the number of observed and simulated infected fish at each sampling point. We let *sse*(θ) be the sum squared error of Y^*^(*t*, θ):

(3) $$\begin{array}{l} sse(\theta )=\sum_{t}{\left(Y(t)-Y^{*}(t,\;\theta )\right)}^{2} \end{array}$$

**Table 1:** Parameters estimates for the SI_E_ compartment model for PMCV in Ireland.

**Parameter**	**Description (unit)**	**Value**
α	Rate of shedding from infected individuals (units per day)	1.0 × 10^0^
β_*q*1_	Decay of environmental infectious pressure in quarter 1 (per day)	9.73 × 10^−2^
β_*q*2_	Decay of environmental infectious pressure in quarter 2 (per day)	8.89 × 10^−2^
β_*q*3_	Decay of environmental infectious pressure in quarter 3 (per day)	1.10 × 10^−1^
β_*q*4_	Decay of environmental infectious pressure in quarter 4 (per day)	9.84 × 10^−2^
*v*_*j*_	Indirect transmission rate of the environmental infectious pressure in juvenile fish (per fish per day)	6.86 × 10^−4^
*v*_*s*_	Indirect transmission rate of the environmental infectious pressure in smolt (per fish per day)	1.64 × 10^−3^
*v*_*g*_	Indirect transmission rate of the environmental infectious pressure in growth and broodstock fish (per fish per day)	1.56 × 10^−2^
D	Spatial coupling of the environmental contamination among proximal nodes (within 10 km) (km/day)	1.11 × 10^−1^

Using the stochastic simulator SimInf, each outcome provided a measurement of the system with process noise, and the average sse, $\bar{sse}\;$, was estimated from *N* = 100 trajectories. The objective function to measure the agreement was then defined as:

(4) $$\begin{array}{l} \bar{sse}=\frac{1}{N}\sum_{N}sse\left(\theta \right) \end{array}$$

The Nelder-Mead algorithm was used to obtain the parameter combination θ that minimized $\bar{sse}$ (35) using a linearly constrained optimisation method in R.

Four parameterization strategies were evaluated based on different combinations of the decay of environmental infectious pressure, β, and the state transition rate, *v*, from susceptible to infected individuals: (1) β was constant across the year and *v* equal across all age categories; (2) β was allowed to vary in each quarter of the year; (3) *v* was allowed to vary across three age group (egg-juvenile, smolt, and growth-repro); and (4) both β and *v* were both allowed to vary, the former by each quarter of the year and the latter by age group. The final parameterization chosen was the one that produced the lowest $\bar{sse}$ (Table 1).

### Specification of Events

In the model, four event types were defined. “Enter” concerns hatchings and international imports. “Internal transfer” occurs on the day that individual fish change their age category, from egg-juvenile to smolt, or from smolt to growth-repro. “External transfer” occurs when fish move from one farm to another. “Exit” is linked with slaughter, euthanasia or international export, and from this point these fish are no longer included in the simulation. Each of the scheduled events was executed in the model once the simulation, in continuous time, reached the time for any of the events. Individuals were sampled at random from the compartments affected by the event. For example, for an external transfer event of *n* smolt from farm 1 to farm 2, *n* smolt were randomly selected from all smolts (including those susceptible and infected) in farm 1 and placed into the same compartments in farm 2.

Fish that entered the model were assumed to be susceptible in their respective age category. Imported fish were assumed to be susceptible, noting the aim of the study to explore spread in Ireland without considering international importation of PMCV. On average, 2,176,111 eggs (range 490,000–5,120,000) were imported per year during the study period, according to information from the Irish Marine Institute (MI). Fish remained in the same infection state whilst changing age category, from egg-juvenile to smolt or smolt to growth-repro, or moving between farms.

Figure 1 presents the conceptual SI_E_ compartment model for PMCV spread in farmed Atlantic salmon, including indirect transmission via the environment and fish movements between holdings.

### Input Data

There is an EU legal requirement for aquaculture production businesses (APBs) to be registered, and to keep records of all movements of aquaculture animals and products, both into and out of the farm (36). In Ireland, the storage of these movement data is undertaken by the MI. This database contains several variables, including the date of fish movement, origin and destination sites with geographic coordinates, life stage, species and quantity of fish moved.

The present study was based on all fish movement reports to the Fish Health Unit of the MI covering the period from 1 January 2009 to 23 October 2017. This included 648 reports, with information about the identifier of the origin farm, identifier of the destination farm, the number of fish and age group (eggs, fry, smolt, growth, broodstock) and the date of the movement. Each record was linked to the geographical coordinates of the farms provided by the aquaculture production business records, to allow for incorporation of local spread during the modeling phase of this study. A farm was considered “active” if any fish were present on the farm, according to movement records.

The following data processing steps were used to generate events for the simulation. Enter events, i.e., hatchings or imports (beyond those officially reported), were imputed as needed to ensure that farm-level fish numbers were sufficient to allow fish shipments between farms as recorded in the fish movement database (i.e., when sent fish were more than those available on the farm at the time based on fish movement records). The date of the imputed hatching events was calculated based on the average residence time of fish prior to shipment in the farm. In total, 90 enter events (73,203,955 fish) were imputed, including approximately a third of these during the first year of the simulation (28 enter events, 10,657,136 fish). This represented on average 10 imputed enter events (8,133,773 fish) per year. Most of this imputed enter events corresponded to eggs (30 events, 48,302,578 fish) or juvenile fish (25 events, 15,946,694 fish), but some fish were entered as smolts (19 events, 6,050,858 fish), growth (15 events, 2,893,825 fish), or broodstock fish (one event, 10,000 fish). Internal transfer events, i.e., moving from egg-juvenile to smolt or from smolt to growth-repro, were imputed when the relevant time during the simulation had been reached. When moving from egg-juvenile to smolt, fish were aged a week prior to shipping to seawater farms, and for aging from smolt to growth-repro, smolts were allowed to remain on the farm for 180 days. Exit events, i.e., mortality, slaughter, or euthanasia, were generated either the day prior to the last shipment of a fish cohort, when it was evident, based on the records, that the fish destination of the whole cohort was another farm (e.g., freshwater farms sending fish to seawater farms, post-smolt seawater farms sending fish to other seawater farms for on-growth), or after a fixed amount of time if it was clear from the records that the farm was the final destination of the fish (e.g., the destination was a seawater farm for on-growth with no record of transfer to another farm). The duration of this period was 300 days in freshwater farms and 600 days for seawater farms. Broodstock fish in freshwater farms were assumed to live until 1 week prior to an egg shipment.

A total of 55 unique farms (the final data set) were used for the simulation, with the following event types: enter, including reported imports (*n* = 135 events, mean = 687,326 affected individuals), internal transfer (*n* = 223, mean = 582,967), external transfer (*n* = 648, mean = 214,330), and exit (*n* = 362, mean = 255,384).

A time-series was created to explore seasonality in the input data, focusing on the number of events, the number of farms with at least one fish and the number of fish per age category. A further time-series was produced to investigate the proportion of farms connected to at least one other farm, for each month of the year. A smoother was added to each of these time-series, using local polynomial regression fitting (loess) in order to describe the temporal trend (37).

### Computational Simulation Framework

The disease spread model was implemented in SimInf (12, 38), which is an R (39) package for data-driven stochastic disease spread simulations. This package was adapted, in part, from the Unstructured Mesh Reaction–Diffusion Master Equation (URDME) framework (40, 41). It interfaces high performance compiled code (including a core algorithm written in C) (42) and OpenMP, which allows work to be divided across multiple processors and computations to be performed in parallel. Implementation and data structures of the simulation algorithm are presented elsewhere (11, 12). The disease spread simulations were performed using the SimInf package version 5.1.0 (model SISe3_sp) and R version 3.4.2.

### Initialization

The simulation was initiated by first supplying the model with an initial state in every farm, together with all events. At the outset, infection prevalence was assumed to be zero, since the earliest detection of the agent in the country, on archived broodstock samples, was in July 2009 (28). The initial environmental infectious pressure, φ_*i*_(0), was also set to zero, as it was assumed that PMCV was not present in the country at the time of initialization (the beginning of January 2009). Based on test results on archived broodstock samples (see Parameter Estimation), the introduction of the agent was assumed to have occurred in two separate occasions at two different farms: 1 month prior to the time of sampling of the first positive archived sample (June 2009) in the population of broodstock fish resident in the farm at the time of sampling, and a second time in November 2009, affecting broodstock fish in a broodstock farm (see Parameter Estimation) where it was later detected in December of that year.

### Sensitivity Analysis

Sensitivity analyses were conducted to evaluate the influence of variation in model parameters on the outcome of the simulation experiment at a national scale. Firstly, the mean time to reach a between-farm prevalence of 50% or more was determined for different values of α, β_*q*1_, β_*q*2_, β_*q*3_, β_*q*4_, ν_*j*_, ν_*s*_, ν_*g*_, and D (the spatial coupling parameter). For each of these parameters, a scaling factor of 0.5–1.5, using steps of 0.1, was used. The following combinations were evaluated during the simulation experiment: each combination of the scaled values of α against the scaled values of β, each combination of the scaled values of α against the scaled values of ν, and each combination of the scaled values of β against the scaled values of ν. Secondly, the evolution of mean farm prevalence over time was determined based on different parameter assumptions regarding distance (distance dependence [euclidean distance, 1/distance^2^ or 1/distance^3^], threshold distance [(8, 10, 15, 20 km]) and seasonality (Quarter: Jan-Mar, Apr-Jun, Jul-Sep, Oct-Dec; Season: 22 Dec-21 Mar, 22 Mar-21 Jun, 22 Jun-21 Sep, 22 Sep-21 Dec; Seasonal sea temperature: Dec-Feb, Mar-May, Jun-Aug, Sep-Nov). For each of the sensitivity analyses, the mean result was determined based on 40 stochastic simulations.

### Exploring Spread on a National Scale

With the final model, farm and fish PMCV prevalence were estimated on a daily basis following the simulated introduction of the agent. The spread of the agent was plotted as a time-series and the modeled epidemic curves were described. The role of both local spread and fish movement was evaluated by setting either *D* (the spatial coupling parameter) to zero (i.e., no local spread), or moving all fish shipped to the susceptible compartment at the time of shipment (which in practice means that all farms will receive fish that are not infected with the agent).

### Evaluation of Control Measures

In addition, we evaluated the effectiveness of an improved biosecurity in specific farms. For the purposes of the simulations, our definition of biosecurity refers to measures that prevent infected fish from entering a farm, akin to a biosecurity strategy that is 100% effective in preventing infected fish from entering or leaving a farm. As described above, this was done by moving all fish shipped to the susceptible compartment at the time of shipment. Six strategies were tested. In the first strategy, all farms were targeted for an increase in biosecurity. This would be a very costly approach, but a good ideal for comparison. In the remaining strategies, we targeted the 8 most central farms in terms of a specific farm centrality measure, which were indegree, outdegree, incloseness, outcloseness, and betweenness, using the same methodology described previously by Yatabe et al. (3). The sample size (ie 8 farms) was chosen arbitrarily, representing ~25% of farms in Ireland at that time. Briefly, indegree describes the number of different farms from which a farm receives fish, outdegree describes the number of different farms to which a particular farm sends fish, incloseness is an estimate of how close all other farms reach to a respective farm, outcloseness is an estimate of how close a respective farm reaches to other farms, and betweenness is a measure of the degree to which a particular farm falls on the shortest path between all pairs of farms in the network (43). For estimating these centrality measures, at the beginning of every year the movement records from the preceding 2 years were used for estimation. For example, on 1 January 2011 centrality measures of all farms were estimated based on fish movement data from 1 January 2009 to 31 December 2010, farms were ranked and the top 8 for each centrality measure were selected for an increased biosecurity. There were two exceptions to this 2-year window for estimation of centrality measures: the year 2010, where only the data from 2009 was used to estimate centrality measures, and 2009, where no data from previous years were available. Therefore, during this latter year no control measures were applied. The former six strategies were evaluated using two approaches for preventing the spread of a newly introduced agent into the country: firstly, by applying the control measures 1 month after the agent was first detected (assuming it was detected on 1 June 2012), from now on the ‘reactive’ approach, and secondly by applying the control measures as a standard practice from before the first detection of the agent (starting on 1 January 2010), from now on the “proactive” approach.

## Results

### Fish Population and Events

**Figure 2:**
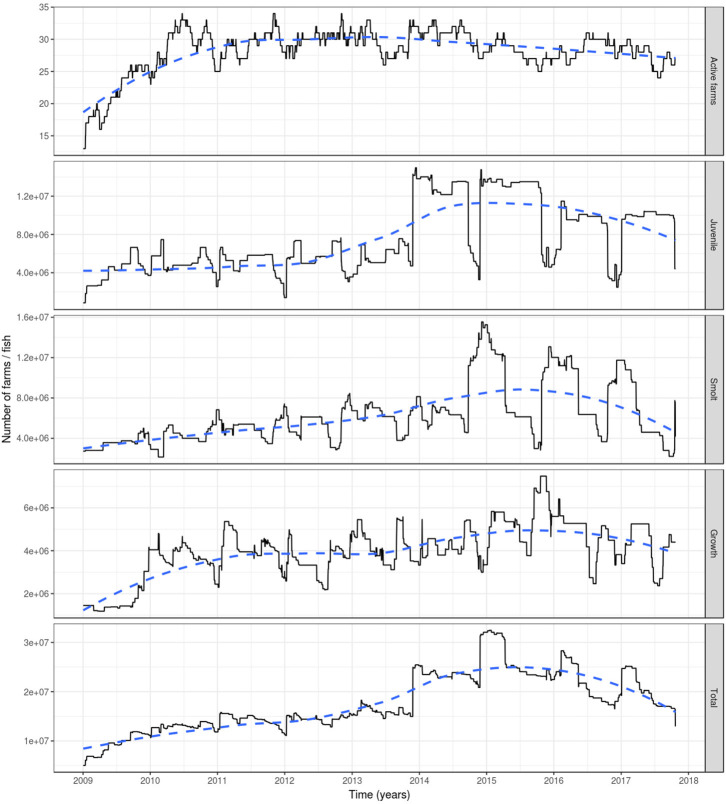
The number of active farms (having at least one fish) (top graph) and of farmed Atlantic salmon (egg-juvenile, smolt, growth-repro, in total; second top to bottom graph) in Ireland during 1 January 2009 to 23 October 2017. Based on fish movement data reported by fish farmers to the Irish Marine Institute. The age categories include egg-juvenile [from egg until 7 days prior to transfer to a marine farm], smolt [from 7 days prior to the transfer to a marine farm to 180 days after the transfer] and growth-repro [more than 180 days in a marine farm]. Dashed line: loess smoother with large span (0.75) to capture long-term patterns.

Based on the data available for 2009, a rapid increase was observed that year in the number of active farms and fish (Figure 2). However, this is an artifact as many farms had not yet been involved in fish movement and thereby appeared inactive (holding no fish). As a consequence, our results are reported from the start of 2010.

The number of active farms declined slightly during the period 2010–2017 with relatively stable numbers (between 25 and 34 active farms) during the 2010-2014 period, and a decrease during the 2015–2017 period (between 24 and 31 active farms) (Figure 2). The total farmed Atlantic salmon population in Ireland had an increasing trend from 2010, with a peak of more than 32 million fish in early 2015, to later decrease until the end of the simulation, although not dropping to previous levels, where it reached ~13 million fish. This increase was related to a large increase in the number of juveniles (specifically eggs) during 2014 and 2015. The number of fish within each age category varied seasonally, with juvenile fish showing peaks during winter and dips during autumn (and to a lesser extent in spring), the former associated with spawning and the latter with the transition of juvenile fish to smolts prior to stocking in seawater farms in autumn and spring. For the smolts, the converse was true, with peaks during autumn and spring. This age group decreases roughly every 180 days, as this is the amount of time after which they were aged into the growth-repro age group. This in turn determines the peaks of this latter age group. The reduction in the numbers of fish in the growth-repro age group were mainly in spring-summer and autumn-winter, being a mixture of elimination of fish stocked in a farm as smolts after 600 days and elimination of fish stocked at older ages after spending the mean residence time in the farm (Figure 2).

**Figure 3:**
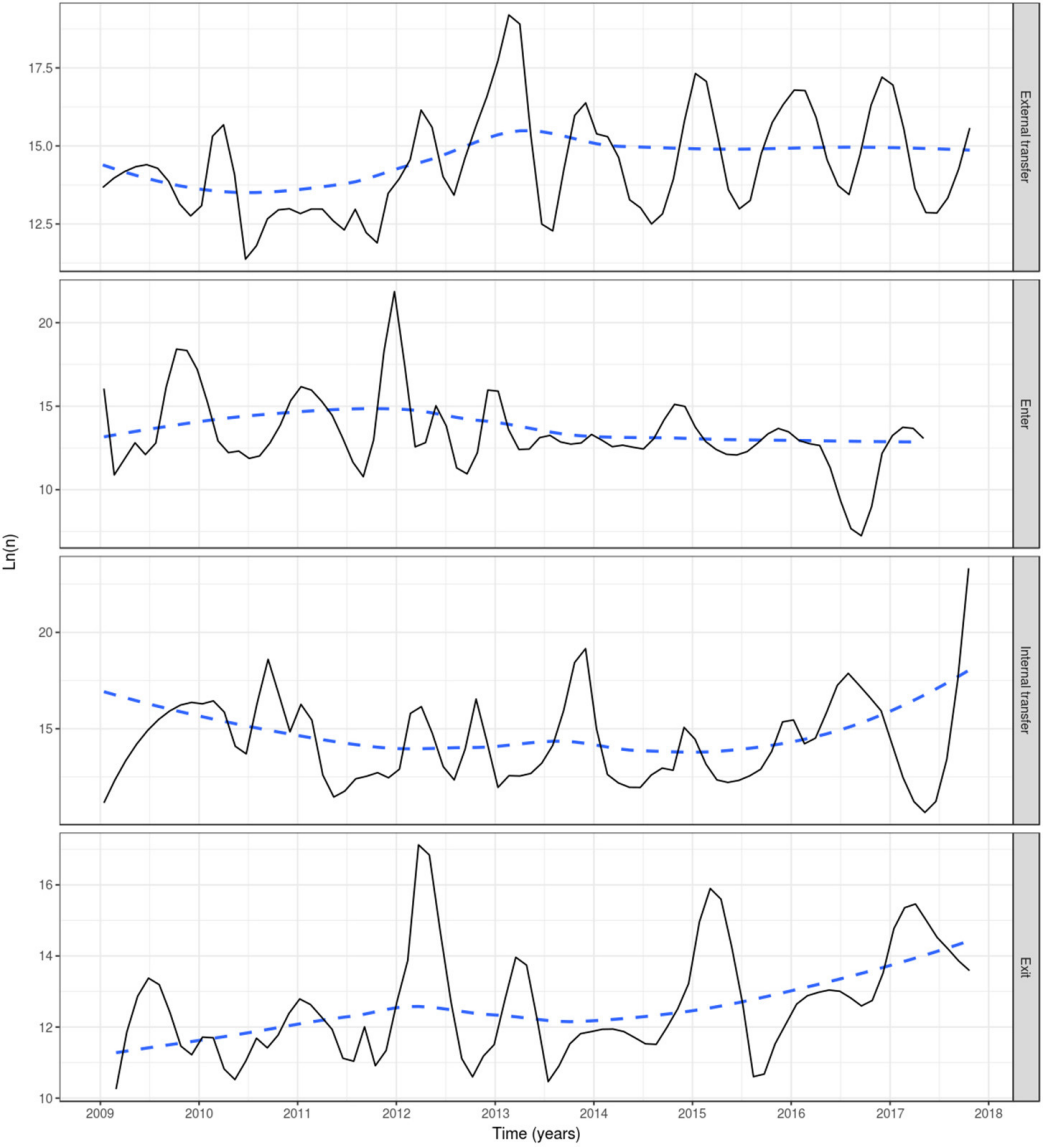
Events in the Irish farmed salmon population, including external transfer, enter, internal transfer and exit (top to bottom). Smoothed (log) number of fish affected by four event types each month in a simulation of PMCV based on fish movement data reported to the Irish Marine Institute by fish farmers during 1 January 2009 to 23 October 2017. “External transfer” occurs when fish move from one farm to another. “Enter” concerns hatchings and international imports. “Internal transfer” occurs on the day that individual fish change age category, from egg-juvenile [from egg until 7 days prior to transfer to a marine farm] to smolt [from 7 days prior to the transfer to a marine farm to 180 days after the transfer], or from smolt to growth-repro [more than 180 days in a marine farm]. “Exit” is linked with slaughter, euthanasia, or international export, and from this point these fish are no longer included in the simulation. Solid line: loess smoother with a small span of (0.1) to capture short-term patterns; Dashed line: loess smoother with large span (0.75) to capture long-term patterns.

**Figure 4:**
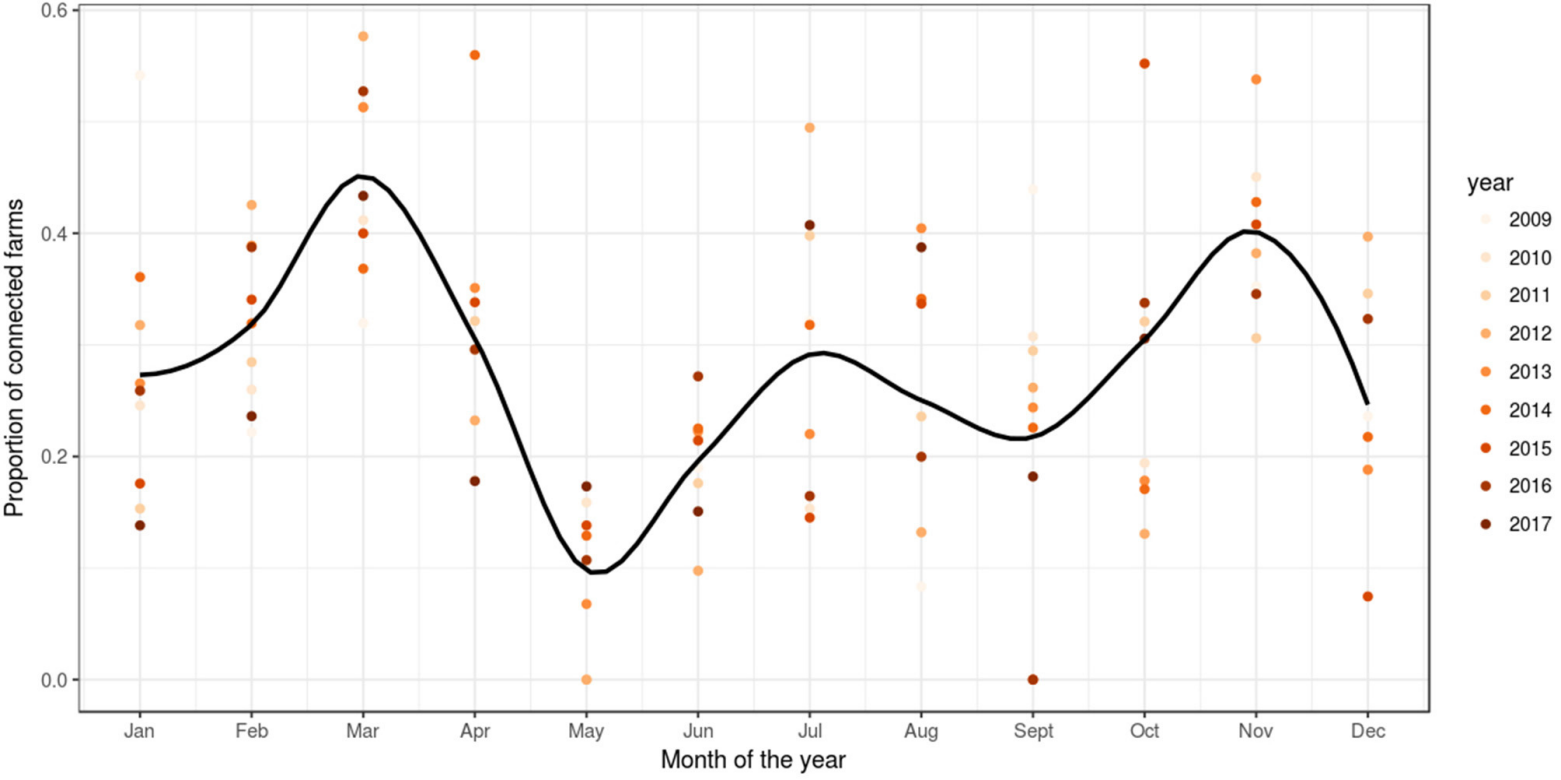
Proportion of farms connected through live fish movements. Scatter plot of the proportion of farms per month of the year with at least one connection to another farm in a simulation of PMCV in the Irish network of live fish movements. The graph is based on fish movements reported to the Irish Marine Institute by fish farmers during the period 1 January 2009 to 23 October 2017. Solid line: loess smoother with a span of (0.4) to capture short-term patterns.

Based on reported fish movement data, the externally scheduled events showed a moderate increase during the study period, except for the enter events, and exhibited seasonal variation. External transfers (i.e., fish shipments) increased from autumn through to spring, decreasing during summer, showing the seasonality in the smolt stocking in autumn and spring, and the spawning season in winter, where fish and eggs are moved from broodstock sites to hatcheries. The enter events (i.e., hatchings and imports) peaked from autumn to winter, this being associated with the entry of fertilized eggs by local broodstock fish (and to a lesser extent imports) and tended to drop during spring and summer. Overall, the enter events showed a moderate decrease during the study period. Internal transfers (i.e., aging to the smolt and growth-repro age groups) had a seasonal pattern in line with the external transfers, as it was often the case that fish will be aged before movement to other farm, specifically fish in the egg-juvenile age group were aged to smolt when moving from a freshwater to a seawater farm, and smolts were aged to growth-repro age group when moving smolt from a seawater to a seawater farm. Exit events (i.e., mortality, exports) also followed external transfers closely. This is because these events were scheduled to occur the day prior to a fish shipment, if the number of fish to be shipped was less than the number of fish initially stocked with the cohort (as no mortality records were available) (Figure 3). The proportion of farms connected with at least one other farm within a month through live fish movements also showed a cyclical pattern, with peaks in February through to April, June through to August, and October through to December (Figure 4).

### Parameter Estimation

Parameter optimization results show that the model with the lowest $\bar{sse}$, a value of 108.2, was the model that allowed indirect transmission rate (ν_*j*_) to vary by age group and decay of the environmental infectious pressure (β) to vary by quarter (the parameter estimates for this model are presented in Table 1). The closest model was the one with varying indirect transmission rate and constant decay of the environmental infectious pressure, with an $\bar{sse}$ of 114.4, while the one with largest $\bar{sse}$ was the model with constant indirect transmission rate and decay of the environmental infectious pressure, with a value of 188.7 (Table 2).

**Table 2:** Mean sums of squared error ($\bar{sse}$) of 4 different parameterizations of a simulation model of PMCV spread in Irish salmon farms. *v* is the indirect transmission rate, β is the rate of decay of the environmental infectious pressure.

**Model**	**Number of estimated parameters**	**$\bar{sse}$**
Constant *v* and constant β	3	188.7
Constant *v* and varying β	6	141.8
Varying *v* and constant β	5	114.4
Varying *v* and varying β	8	108.2

**Figure 5:**
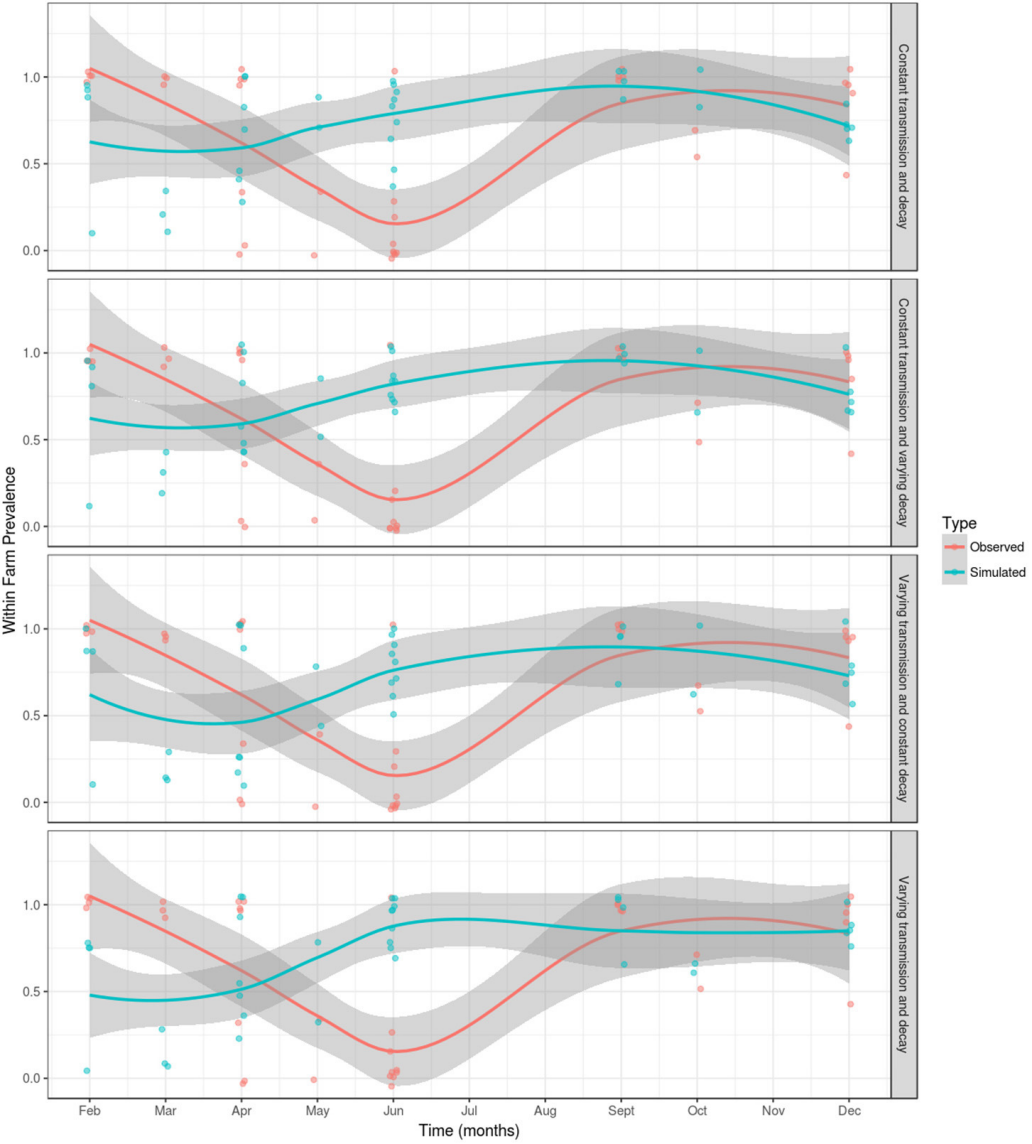
A comparison of observed and simulated within-farm PMCV prevalence in the Irish farmed Atlantic salmon population during 1 January 2009 to 23 October 2017. Within-farm PMCV spread was modeled using a SI_E_ compartment model with the two disease states: susceptible (S) and infected (I). E represents the environmental compartment contaminated with PMCV by infected fish and its local spread to neighboring farms. Comparison of the observed proportion of PMCV positive fish sampled per farm per date in a longitudinal observational study of the PMCV status (28) with simulated status. Each figure shows the sample prevalence values for each month as (jittered) dots and a loess with a span of 0.75 with a 95% confidence interval for observed and simulated data. A jitter was added to the points for better visualization.

For each of the model parameterizations, the simulated outcome did not show a seasonal variation in the proportion of PMCV positive sampled fish, even when decay of the environmental infectious pressure was allowed to vary. A comparison of the results from the observational study (28) and the simulated outcome from each model parameterization is presented in Figure 5. For all model parameterizations, the results from the month of June are poorly fitted, however, the model fit is better from September through to December and February through to May. The remaining results will refer to the best fitting model (i.e., varying *v*_*j*_and varying β).

### Sensitivity Analysis

The sensitivity analysis results are presented in Images S1, S2. Analyses were performed over the required time to reach a between-farm prevalence of 50% or more when scaling model parameters by factors of 0.5 through 1.5 by 0.1 increments. The model is sensitive to changes in the indirect transmission rate (i.e., *v*, upsilon), decay of the environmental infectious pressure (i.e., β, beta), and the rate of viral shedding from infected individuals (i.e., α, alpha). The model did not show great sensitivity to changes in D, the spatial coupling parameter, indicating that local spread is not a very important mechanism of viral transmission. Using a distance threshold of 10 km, the of the model using euclidean distance (108.2) was better than for either 1/distance^2^ (158.9) or 1/distance^3^ (167.2). There were minimal differences in the evolution of farm prevalence over time based on different distance cutoffs and seasonality intervals (Image S2).

### Exploring Spread on a National Scale

**Figure 6:**
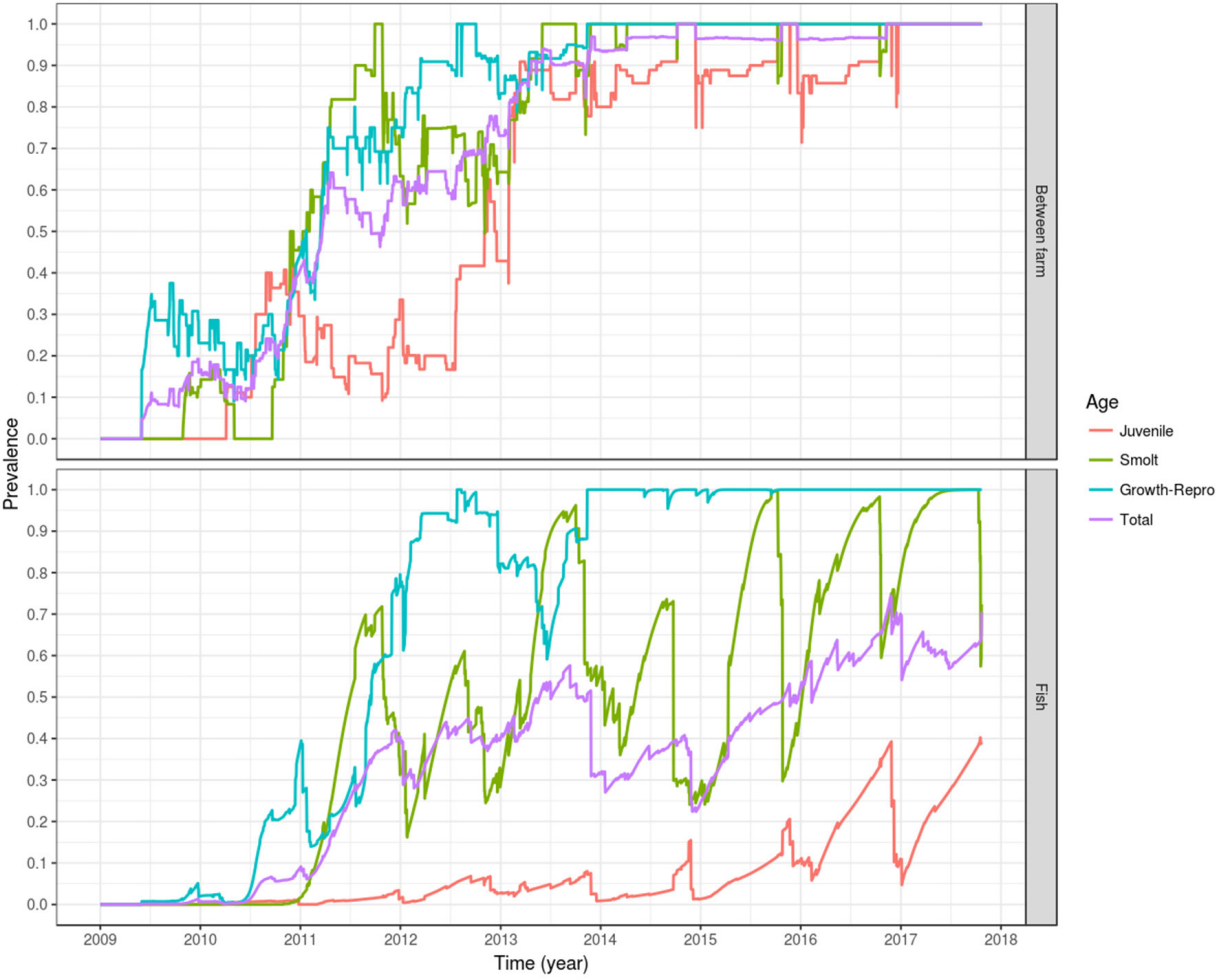
Prevalence of simulated PMCV infection dynamics. The plotted values correspond to the mean of the outcome from 100 stochastic simulations of the spread of PMCV in the Irish farmed Atlantic salmon population during 1 January 2009 to 23 October 2017. Within-farm PMCV spread was modeled with a SI_E_ compartment model with the two disease states: susceptible (S) and infected (I). E represents the environmental compartment contaminated with PMCV by infected fish and its local spread to neighboring farms. Top: between-farm prevalence; bottom: fish prevalence.

Following introduction of infection in mid and late 2009, i.e., the index cases, a between-farm prevalence of 50% was reached on early 2011 (<2 years after the first introduction in May 2009), and 90% of the fish farms were infected by early 2013 (<4 years after the simulated introduction). By 5 years after the simulated introduction in late 2014, 100% of the farms were infected, oscillating around this value until the end of the simulation. The farms holding growth fish and broodstock (age group 3) and smolts (age group 2) had a faster modeled epidemic curve, the first group reaching a between-farm prevalence of 50% by early 2011 and 100% by mid 2012, stabilizing around that value in late 2013. For the farms holding smolts, a between-farm prevalence of 50% was reached in late 2010, and 100% between-farm prevalence was reached for the first time in late 2011, dropping to 50–75% during most of 2012, to finally oscillate around 100% from mid 2013 onward. The modeled epidemic curve for farms holding eggs and juvenile fish (age group 1) was slower, reaching a between-farm prevalence of 50% by late 2012, and a between-farm prevalence around 90% since early 2013, oscillating around this value until the end of 2016, where it stabilized at 100% until the end of the simulation (Figure 6, top).

Fish prevalence follows a similar dynamic, with total and juvenile fish prevalence lagging behind fish prevalence in the growth-broodstock and smolt age groups, but with the former two age groups never reaching a level of 100%. This is due to the constant input of newly hatched fish, which the model assumes are introduced into the susceptible compartment. This can be seen as a drop in the egg-juvenile and total fish prevalence around winter, when the fertilized eggs and juvenile fish are entered into the fish population. A similar cyclical trend can be seen with fish prevalence in smolt, which declines in autumn and spring as juvenile fish are transitioning into this stage prior to the stocking in seawater farms. For growth and broodstock, fish prevalence stabilizes around 100% from late 2013 until the end of the simulation (Figure 6, bottom).

### The Role of Local Spread and Fish Movement

**Figure 7:**
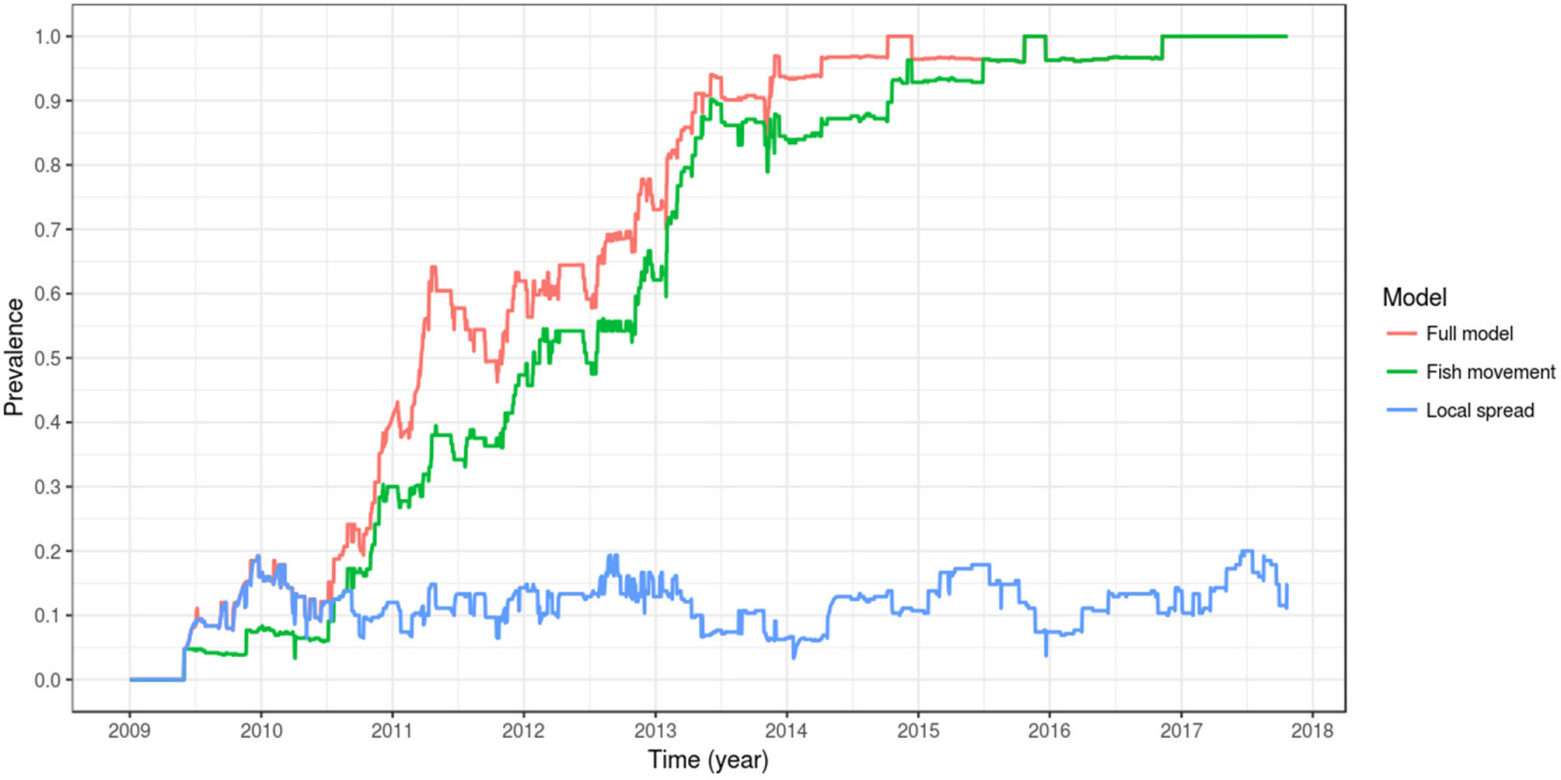
Comparison of between-farm PMCV prevalence dynamics between the full model, a model with spread only via fish movement, and a model with spread only via local spread. The plotted values correspond to the mean of the outcome from 100 stochastic simulations of the spread of PMCV in the Irish farmed Atlantic salmon population during 1 January 2009 to 23 October 2017. Within-farm PMCV spread was modeled with a SI_E_ compartment model with the two disease states: susceptible (S) and infected (I). E represents the environmental compartment contaminated with PMCV by infected fish and its local spread to neighboring farms.

When the effect of local spread was removed from the simulation (i.e., D, the spatial coupling parameter, equal to zero), a model that includes spread between farms is only possible via fish movement (a model with spread only via fish movement). In this scenario, the spread of PMCV slows down, but the overall pattern remains of an increasing trend reaching a between-farm prevalence of 100%. This level of prevalence was reached for the first time in late 2015, compared to the full model where 100% prevalence was reached 1 year earlier in 2014. For the model where transmission was only possible via local spread (where all fish transferred were moved to the susceptible state at the time of each shipment), between-farm prevalence never reached a 20% level until mid 2017, and oscillated most of the time around 10%. Under this scenario, the only time that between-farm prevalence is higher than in the scenario with spread only via fish movement (and very similar to the full model) is at the beginning of the epidemic (mid 2009 to mid 2010), indicating that local spread was the main driver of the transmission between farms at this early time (Table 3, Figure 7).

**Table 3:** Comparison of time to reach prevalence benchmarks at the between-farm level for models with and without local spread.

**Prevalence**	**Full model**		Fish movement alone^*^		Local spread alone^**^	
	**mean**	***SD***	**mean**	***SD***	**mean**	***SD***
10%	220.7	33.9	568.0	0.00	245.3	23.4
20%	593.0	0.0	676.7	6.0	3,092	0
30%	688.5	6.0	758.4	58.1	NA	NA
40%	723.4	20.4	993.0	88.8	NA	NA
50%	808.8	5.5	1,112.6	18.1	NA	NA
60%	832.7	0.5	1,410.2	26.6	NA	NA
70%	1,375.6	45.9	1,493.5	5.2	NA	NA
80%	1,470.1	36.1	1,556.3	13.5	NA	NA
90%	1,570.1	4.2	1,980.2	231.0	NA	NA
100%	2,107.0	0.0	2,487.0	0.0	NA	NA

NA, not applicable;

*, D the spatial coupling parameter, set to zero;

**, *all fish sent to susceptible compartment when shipments are scheduled*.

*The mean times and standard deviations (SD) are based on 100 simulations of the spread of PMCV in the Irish farmed Atlantic salmon population during 1 January 2009 to 23 October 2017. Within-farm PMCV spread was modeled with a SI_E_ compartment model with the two disease states: susceptible (S) and infected (I). E represents the environmental compartment contaminated with PMCV by infected fish and its local spread to neighboring farms*.

### Evaluation of Centrality Based Control Measures

**Figure 8:**
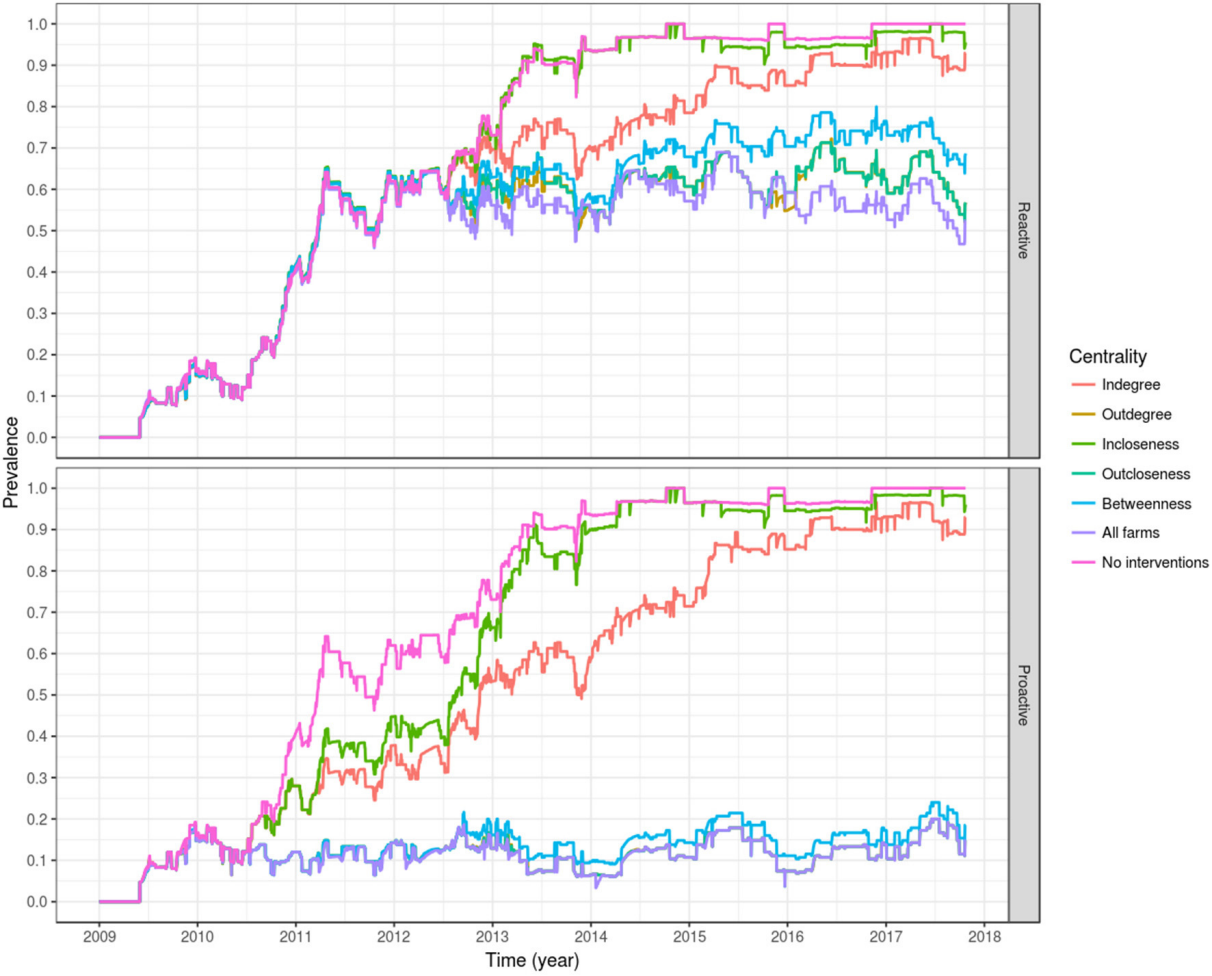
Evaluation of interventions based on farm centrality measures of degree, closeness, and betweenness, applied in a reactive way, 1 month after the first report of PMCV (top), or proactively from 2010 as a permanent policy (bottom). For the proactive approach, the lines of outdegree, outcloseness and targeting all farms are virtually indistinguishable.

Of the evaluated centrality based interventions, the most effective were the ones based on outdegree and outcloseness, for both the reactive and proactive approaches (Tables 4, 5, Figure 8). For the former, after all spread via fish movement from the targeted nodes (based in either node centrality measure) is stopped in July 2012, the increasing trend in between-farm prevalence immediately stops, stabilizing around 60% until the end of the simulation for both outdegree and outcloseness based interventions. The between-farm prevalence obtained with these interventions was slightly higher than the prevalence obtained when pathogen spread via fish movements from all fish farms was stopped, the difference being clearer from 2016 until the end of the simulation (Figure 8 top). In terms of the time required to reach set prevalence benchmarks, both outdegree and outcloseness based targeted interventions are virtually indistinguishable, with the former being slightly better (Table 4). Regarding the targeted interventions based on the other centrality measures, the one based on incloseness was the one that performed worst, with virtually the same result as when no intervention is applied, followed by the ones based on indegree and betweenness, with the latter being similar to the ones based on outdegree and outcloseness until early 2014, after which it produces a higher between-farm prevalence.

**Table 4:** Comparison of the mean time to reach prevalence benchmarks at the between-farm level for models with different farm centrality based reactive intervention strategies.

**Prevalence**	**Full model**	**Local spread**	**Indegree**	**Outdegree**	**Incloseness**	**Outcloseness**	**Betweenness**
10%	220.7	217.3	232.4	233.5	234.9	236.4	232.7
20%	593.0	593.0	593	593	593	593	593
30%	688.5	688.6	684.4	683.8	684.8	684.0	684.6
40%	723.4	726.3	715.8	713.8	718.7	714.7	714.6
50%	808.8	808.2	809.6	812.2	811.3	810.3	810.4
60%	832.7	832.8	842.7	833.6	837.9	835.6	844.9
70%	1,375.6	NA	1,361.0	NA	1,354.2	NA	1,789.9
80%	1,470.1	NA	2,028.2	NA	1,489.3	NA	NA
90%	1,570.1	NA	2,287.7	NA	1,567.6	NA	NA
100%	2,107.0	NA	NA	NA	2,107	NA	NA

*NA, not applicable*.

*The mean times are based on 100 simulations of the spread of PMCV in the Irish farmed Atlantic salmon population during 1 January 2009 to 23 October 2017. Within-farm PMCV spread was modeled with a SI_E_ compartment model with the two disease states: susceptible (S) and infected (I). E represents the environmental compartment contaminated with PMCV by infected fish and its local spread to neighboring farms*.

**Table 5:** Comparison of mean time to reach prevalence benchmarks at the between farm level for models with different farm centrality based proactive intervention strategies.

**Prevalence**	**Full model**	**Local spread**	**Indegree**	**Outdegree**	**Incloseness**	**Outcloseness**	**Betweenness**
10%	220.7	235.7	231.8	228.3	230.0	231.7	231.0
20%	593.0	3,092.0	593.0	3,092.0	593.0	3,092.0	1,353.8
30%	688.5	NA	750.7	NA	736.8	NA	NA
40%	723.4	NA	1,260.9	NA	851.3	NA	NA
50%	808.8	NA	1,413.0	NA	1,312.1	NA	NA
60%	832.7	NA	1,530.8	NA	1,408.2	NA	NA
70%	1,375.6	NA	1,923.4	NA	1,435.7	NA	NA
80%	1,470.1	NA	2,258.9	NA	1,546.0	NA	NA
90%	1,570.1	NA	2,642.0	NA	1,686.3	NA	NA
100%	2,107.0	NA	NA	NA	2,107.0	NA	NA

*NA, not applicable*.

*The mean times are based on 100 simulations of the spread of PMCV in the Irish farmed Atlantic salmon population during 1 January 2009 to 23 October 2017. Within-farm PMCV spread was modeled with a SI_E_ compartment model with the two disease states: susceptible (S) and infected (I). E represents the environmental compartment contaminated with PMCV by infected fish and its local spread to neighboring farms*.

Similarly, when targeted interventions are applied from early on in the proactive approach (from January 2010), the most effective targeting strategies are those based on outdegree and outcloseness, which are virtually indistinguishable from the one based in removing spread via fish movement from all nodes. The strategies based on these centrality measures produced between-farm prevalences around 10% from their implementation through the end of the simulation. Similar to the reactive approach, the worst performing strategy here is the one based on incloseness, which produces virtually the same result as if no intervention was applied, although with a slight delay in the increase of between-farm prevalence from 2010 through mid 2014. The strategy based on indegree was second to last, reaching a between-farm prevalence of around 90% in early 2016 and stabilizing around that value until the end of the simulation. A betweenness-based strategy did not show a clear difference with the best performing strategies based on outdegree and outcloseness until late 2012, where between-farm prevalence increased slightly above the value for the other two strategies, and this difference remained until the end of the simulation (Table 5, Figure 8 bottom).

## Discussion

In this paper, we describe the use of data-driven network modeling as a framework to evaluate the transmission of PMCV in the Irish farmed Atlantic salmon population, and the impact of targeted intervention strategies. To do this, we have simulated the introduction and spread of PMCV in the Irish Atlantic salmon farming industry using real data of live fish movements, compulsorily reported to local authorities during 1 January 2009 to 23 October 2017, and data from a prevalence study conducted from 30 May 2016 to 19 December 2017. Additionally, using the fish movement data set, we have imputed population dynamics events at the farms (hatchings-imports, aging, and deaths-exports) by using a set of rules based on domain knowledge of the fish production cycle. We were able to reproduce population dynamics and the observed PMCV prevalence in the observational study that was used to estimate model parameters, evaluate the importance of infection spread via fish movement and local spread, and evaluate the effects of different farm centrality based control strategies.

Parameter estimation showed that the best fitting model was the one with increasing transmission rates as fish aged and with a rate of decay of the environmental infectious pressure that varied each quarter (Tables 1, 2). In common with other viral infections of farmed Atlantic salmon, studies have shown that fish have increased PMCV prevalence and higher concentration of the virus in fish tissues as they age during the production cycle. Further, the probability of developing CMS increases with the length of time at sea (44). In the freshwater phase, viral particles are detected in low quantities, and CMS outbreaks and CMS-related pathological lesions have not been described (32). In a study to evaluate vertical transmission of the agent, PMCV was found in 128 of 132 broodfish, and later detected in all stages of progeny, but only at prevalences of <25% and with concentrations close to the detection limit of the method (25, 26). In the observational study used for estimating the parameters in our model, PMCV was found at higher concentrations in broodstock fish and lower concentrations in younger age groups (28). Although pathogen concentration was not part of our model, a possible extension would be to allow α, the rate of viral shedding, to vary by age group.

In our modeling, simulation was initialized at two broodstock farms. Within these farms, transmission was horizontal (fish are entered into the model as susceptible, noting that vertical transmission is not considered). As highlighted in the model, horizontal transmission between farms is important, but only via fish transfer and not via local spread. Our results indicate that the introduction of the agent in two specific farms during the second half of 2009, coupled with the structure of the network of live fish movements in the country, is enough to account for the widespread occurrence of PMCV currently observed in the country. These findings are in agreement with the recent work of Tighe et al. (29), who found that PMCV strains in Ireland are largely homogenous, without evidence of geographically linked clustering, consistent with a hypothesis of agent spread through fish movement (that is, the Irish industry as a single epidemiologically linked unit). If local spread were the main driver, several locally distinct viral strains would be more likely. In addition, Tighe et al. (29) suggests that the Irish strains from cluster I could have arrived in Ireland between 2010 and 2012, while the strains from cluster IV could have arrived between 2007 and 2009. This is very close to our simulated introduction during 2009 based on the results of archived samples. This study also suggests that these dates are supported by the testing of archived heart samples from Irish Atlantic salmon broodstock (28) which showed that all samples collected prior to 2009 were PMCV negative, whereas those tested from 2009 onwards were positive. It is these data, from Morrissey et al. (28) that form the basis for the current simulation study.

PMCV is observed at low levels during the freshwater phase. It is not known whether this virus can persist in these fish throughout the production cycle. Further, it is unclear whether persistent virus in these fish is a substantial contributor to mortality at sea compared to the infection pressure that is exerted from neighboring farms and other factors, external to the farm, that are associated with infection and disease (25). In recent work, Jensen et al. (27) have highlighted a possible pathway of transmission from broodstock to smolt, a pathway that is not explicitly modeled in the current study. We consider that our modeling approach would be well-suited to evaluate the plausibility of alternative transmission routes.

Although current parameter estimates appear to reproduce age-varying fish susceptibility, it was not possible to reproduce the observed drop in prevalence during the May-July period. There are reports of slight seasonal variations of clinical CMS in seawater farms, with an increase in cases in autumn and spring (45), but no reports on seasonal patterns in the detection of PMCV via RT-PCR or other diagnostic tests, let alone seasonality of detection in freshwater. The fact that all model parameterizations used were not able to reproduce the observed drop during the month of June leads us to think that further observational data is required, possibly with a study with sampling conducted evenly throughout the year, so it can include the months where no samples were taken (July, August, and November) and a more homogeneous number of farms (ideally the same throughout the study) and fish sampled at each time. Nonetheless, we believe that our model is valuable, and that important lessons could be learned from it, like the major importance of spread via fish movement and the best intervention strategies in order to prevent extensive infection spread. These lessons would apply not only to PMCV, but also to infectious diseases whose spread is predominantly via fish movement (5, 6, 46–52).

The decision to use a susceptible-infected (SI) over a susceptible-infected-susceptible (SIS) model for within-farm spread was based on the fact that different experimental studies have found the viral genome present in tissues of challenged fish throughout the whole duration of the study, indicating that the salmon immune response may be unable to eliminate the virus (32–34). This, together with studies where PMCV has been consistently found in cohorts of fish sampled through long periods of time, indicating that PMCV can be present in fish for some months (26, 53), provides further support for the modeling approach used here. Nevertheless, more research is required to further validate or refute this modeling choice, as it is possible that fish clear the infection beyond the time frames used in both experimental and observational studies.

The model was sensitive to changes in the values of the indirect transmission rate, rate of decay in environmental infectious pressure, and the rate of viral shedding from infected individuals, but not to changes in the level of spatial coupling (Image S1). Model outputs were also not substantially influenced by different parameter assumptions regarding either distance (distance dependence, threshold distance) or seasonality (Image S2), noting that information about distance thresholds was derived from other viral infections such as infectious salmon anemia (ISA), where estimates have varied from 5 to 20 km or more (54–56). Collectively, these results suggest that local spread may play a secondary role in the spread of PMCV across the Atlantic salmon farms in the country. When local spread was removed completely from the model (Table 3, Figure 7), it was even clearer that this transmission pathway under current model assumptions was not the most important. On the basis of these results, we hypothesize that the widespread presence of PMCV in Ireland is most likely a product of the shipments of infected but subclinical fish through the network of live fish movements that occur in Ireland. This is consistent with fish being infected but subclinical for months prior to manifesting signs of disease (26), and by the structure of the network of live fish movements in the country (presence of highly connected hubs, short average path lengths, and movements of fish that encompass the whole country) (3).

There is limited knowledge of agent survival of PMCV in the aquatic environment. Infection risk is higher on farms with a history of CMS outbreaks (44), which could suggest survival of the causal agent in the local environment. Further, infection pressure from farms within 100 km of seaway distance was found to be one of the most important risk factors for clinical CMS diagnosis (44), although this study did not evaluate spread via fish movement. It is noted that the distance over which infection can be transmitted via water is determined by an interaction between hydrodynamics, viral shedding and decay rates (46). Further research on PMCV survival in the environment is needed to guide parameterization of future models.

The most effective intervention strategies are based on outdegree and outcloseness (Tables 4, 5, Figure 8), with the highest impact being observed when using these intervention strategies with a proactive approach (Table 5, Figure 8 bottom). Note that all outgoing shipments from selected farms are assumed to include only susceptible fish (that is, no infected fish), which can be equated with high levels of biosecurity. The outdegree and outcloseness based strategies are comparable, most likely because both strategies refer to outgoing shipments from a farm (a farm's influence), the former with the number of farms receiving fish from a given source, and the latter inversely related to the number of intermediaries between the source and the rest of the farms in the network. Both centrality measures were moderately correlated with each other, with a Pearson correlation of 0.53 (*p* < 0.001) for the proactive approach when including all farms for each time window used. Based on a closer examination of the top eight farms of each list, for every year (except 2013 and 2016), one list always included at least the top three elements of the other. In other words, each list contained the top three farms in terms of outdegree and the top three farms in terms of outcloseness. Further iterations of this model could exploit the similarity between ranks of farms based on these two centrality measures, for example evaluating the effect of targeting a lower number of farms based on a list created from the top elements of both rankings. For the case presented here (targeting the top 8 farms based on their centrality measures), either centrality measure could be used. Being this the case, we would advocate for the use of outdegree over outcloseness, given its simplicity of estimation and understanding.

The proportion of farms connected via live fish movements varied in a cyclical manner, with spikes during the periods of January-April, July, and October-December, which is consistent with results from our previous descriptive study of the network of live fish movements in Ireland (3). Interventions could be considered that specifically apply at these times of higher connectivity between farms, to take account of this observed cyclicity.

The remaining between-farm prevalence levels observed after the implementation of this targeted strategies is due to residual infectious pressure and local spread, where PMCV is not fully cleared from the environment between generations of fish, allowing its transmission to newly stocked fish and locally between neighboring farms. Similarly, the lower performance of the reactive approach, even if all transmission via fish movements is halted (where between-farm prevalence remains stable at around 60%) suggests that eradication of PMCV is virtually impossible in Ireland, as it seems that after elimination of transmission via fish movements, the agent is consistently sustained by local spread (Figure 8, top).

The lack of complete production records for all Irish Atlantic salmon farms was the main reason for using movement records to recreate fish population dynamics. Nevertheless, we consider that the rules as applied in this study were realistic. For example, if a farm ships fish in excess of the total fish population at the time of the shipment, it is reasonable to assume that these fish must have originated at a previous time. The options for this origin are either non-recorded, incoming fish shipments or hatching (enter events) of new fish. In the case of the latter, this is perfectly reasonable if the fish deficit at the farm is due to a shipment of eggs. However, if the deficit is due to a shipment of older fish (i.e., juvenile, smolt, growth, or broodstock), assigning an enter event for this age groups is not realistic. Nevertheless, in the absence of records accounting for the origin of fish sent in these age groups, this seemed like a better approach than arbitrarily imputing their origin to another farm, which in turn would have created fish deficits in other farms cascading to the rest of the network. Arguably, the availability of complete production records from all Irish salmon farms would minimize this issue, although making such records available for a 9-year time period would pose a hefty burden on fish farmers. Additionally, we assert that the impact of our imputation is marginal, considering that only 90 enter events were imputed during the study period (compared to the observed 648 fish shipments), mostly at the beginning of the simulation (to allow the farms to populate, due to lack of previous years' data), and involving mainly fertilized eggs in freshwater farms. This is further evident when evaluating the generated population dynamics, like the number of fish in each age group (Figure 2) and the timing of fish enter events (Figure 3), where the abundance of each age group and the enter events follow a seasonal pattern that would be expected given the life cycle of farmed Atlantic salmon. Assigning exit events (most of them representing fish mortality) the day before the last fish shipment of a fish cohort was a simplification necessary for allowing farms not to overpopulate as the simulation proceeded. The impact of assuming all fish within a cohort were present until the day before shipping is hard to gauge, but we think it would be a small effect, especially considering the large fish populations involved in salmon farming. Future iterations of this model could include a mortality function fitted from the data, or even better, real mortality data from fish farm production records, if available.

One of the assumptions of the intervention strategies used in this study is that they are 100% effective in eliminating transmission between farms via fish movements. In order to achieve a similar level of effectiveness in the field, it would require screening of all fish shipments with a highly sensitive test before they exit the origin farm, and elimination of all positive batches (possibly after a confirmatory test). The sensitivity of currently used diagnostic methods is not reported in the literature, but one could arguably assume that the RT-PCR method for detection of the virus has a high sensitivity (above 90%) given its capacity to measure viral RNA, which may or may not be present within a virion that is able to replicate. Currently there are no confirmatory tests for PMCV, and diagnosis of the clinical disease is based on clinical observations, necropsy, and histopathological findings (19). As for diagnosing latently or subclinically infected fish, this would pose a great challenge today, as there are no cell cultures or other methods that could assist in such a task, which is particularly important for the correct diagnosis of the agent on the early stages of fish life, namely eggs, juvenile fish, and smolts.

Further, even if accurate diagnostic tests were available, the feasibility of discarding all infected fish consignments is doubtful, as it would impose a heavy burden on fish farmers, especially considering the modeled current levels of PMCV prevalence. This indicates that for PMCV it is already too late for this type of action to be taken. Nonetheless, it does suggest a clear path to prevent the spread of exotic infectious agents in Ireland, such as ISA virus, piscine reovirus (PRV), and others. For these agents, targeted surveillance strategies could be implemented based on the top ranked farms in terms of outdegree as described above, which would allow for a timely detection and prevention of further spread across the country.

In conclusion, in this study we highlight the importance of human-assisted live fish movement for the dissemination of PMCV across the country, and demonstrate a means, using centrality based targeted surveillance strategies, to prevent this type of spread in the future for other infectious disease agents. These strategies should be applied early on in the epidemic process, before country-wide dissemination of the agent has taken place. The Irish salmon farming industry would benefit from this approach, as it would help in the early detection and prevent the spread of exotic viral agents which have the potential to severely impact local farms and the livelihoods of people that depend on them. This in turn would make Irish salmon farming a more robust and sustainable industry, capable of dealing with infectious agents in a timely and effective way, minimizing socio-economic and environmental losses, and maximizing fish health and welfare.

## Data Availability Statement

The datasets generated for this study will not be made publicly available. The movement data are the property of the Marine Institute and the Irish farmed salmon industry, and these organizations would need to provide permission prior to data sharing. Requests to access the datasets should be directed to the Marine Institute (to datarequests@marine.ie or online through www.marine.ie) and to Catherine McManus (Catherine.McManus@mowi.com).

## Author Contributions

TY and BM-L conceived the study. Data collection was undertaken by FG, NR, TM, and CM. TY assembled and validated the data, conducted the literature review, constructed and validated the model, and ran the model scenarios, under the supervision of BM-L, AH, and SM. TY completed the initial draft of the manuscript, which SM subsequently revised and adapted. JD-C conducted further sensitivity analyses. All authors contributed to the article and approved the submitted version.

## Conflict of Interest

CM is an employee of Mowi Ireland (formerly known as Marine Harvest Ireland). The remaining authors declare that the research was conducted in the absence of any commercial or financial relationships that could be construed as a potential conflict of interest.

## Supplementary Material

Image S1 Sensitivity analysis of the mean time to reach a between-farm PMCV prevalence of 50% or higher given different scaling factors for the model parameters. Of interest was the first time that 50% or more of the farms had at least one infected fish based on simulations of PMCV spread in the Irish farmed Atlantic salmon population during 1 January 2009 to 23 October 2017. Time is represented in color [red [733 days] through to blue [1,060 days]]. The contours represent the time taken to reach 50% prevalence for a list of combinations of the x and y parameters. So, for example, a single line represents 900 days to reach 50% prevalence, which can be achieved by having the values of x and y shown in the plot. Parameter estimates were scaled from 0.5 to 1.5, by 0.1, including the indirect transmission rate parameters (v_*j*_,v_*s*_,v_*g*_, i.e., upsilon), decay of environmental infectious pressure (β_*q*1_, β_*q*2_, β_*q*3_, β_*q*4_, i.e., beta), the spatial coupling parameter (D, i.e., coupling), and the rate of viral shedding from infected individuals (α, i.e., alpha). The figure presents 6 different combinations of scaled parameters (e.g., the top left presents the impact on mean time to reach a between-farm prevalence of 50% or higher given different scaling factors for the decay of environmental infectious pressure (beta; on the x-axis) and the indirect transmission rate parameters (upsilon; on the y-axis). For each parameter combinations, the mean time to reach a between-farm PMCV prevalence of 50% or higher was calculated from 40 stochastic simulations.

Image S2 Sensitivity analysis of the evolution of mean farm PMCV prevalence over time given different inputs for distance **(a)**, left, and seasonality **(b)**, right. The distance inputs included distance dependence [euclidean distance [A], 1/distance^2^ [B] and 1/distance^3^ [C]] and distance threshold value (8, 10, 15, 20 km). Therefore, “*t* = 8 km, distance A” refers to the use of euclidean distance during modeling with a distance threshold of 8 km, and “*t* = 20 km, distance B” refers to the use of 1/distance^2^ during modeling with a distance threshold of 20 km. The seasonality inputs included quarter (Jan-Mar, Apr-Jun, Jul-Sep, Oct-Dec), season (22 Dec-21 Mar, 22 Mar-21 Jun, 22 Jun-21 Sep, 22 Sep-21 Dec), and seasonal sea temperature (Dec-Feb, Mar-May, Jun-Aug, Sep-Nov). The plotted values correspond to the mean farm PMCV prevalence during 1 January 2009 to 23 October 2017, based on 40 stochastic simulations.
