# Supplementary figures and images for: Data-Driven Network Modeling as a Framework to Evaluate the Transmission of Piscine Myocarditis Virus (PMCV) in the Irish Farmed Atlantic Salmon Population and the Impact of Different Mitigation Measures
Source: Front Vet Sci. 2020 Jul 16;7:385. doi: 10.3389/fvets.2020.00385 (PMC7378893; doi:10.3389/fvets.2020.00385)

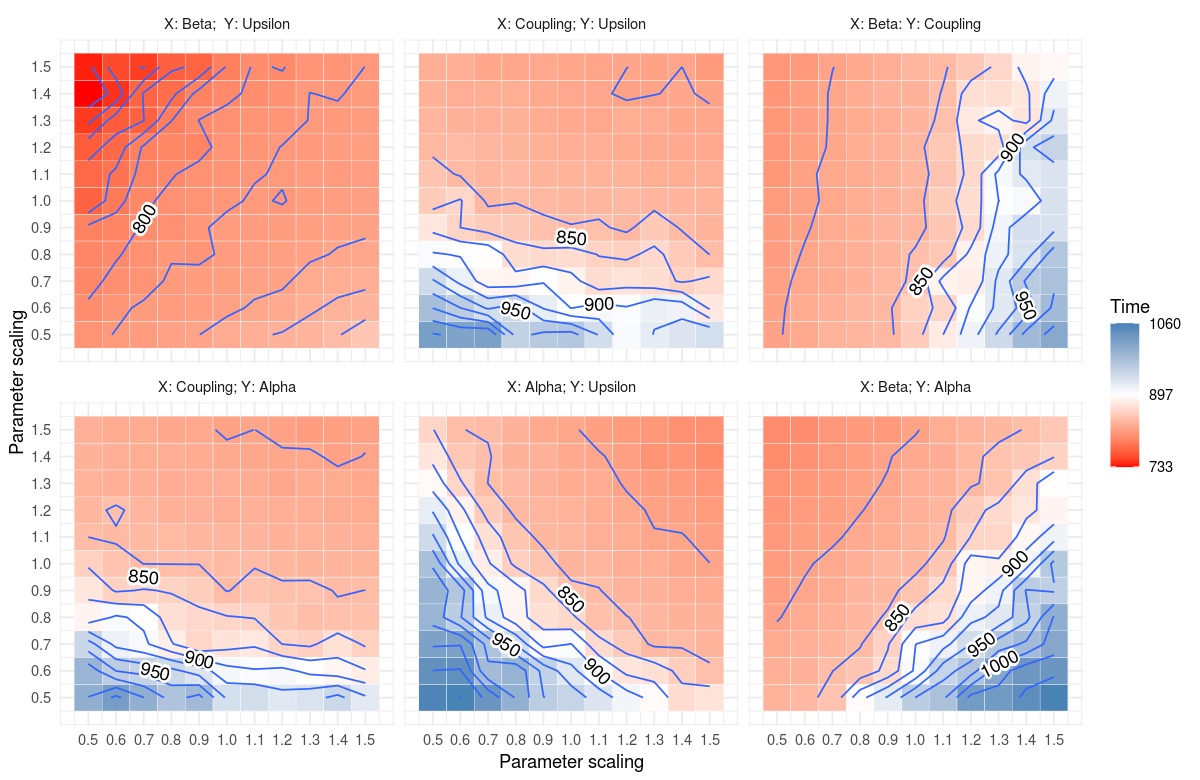

Supplement: Image S1 — Sensitivity analysis of the mean time to reach a between-farm PMCV prevalence of 50% or higher given different scaling factors for the model parameters. Of interest was the first time that 50% or more of the farms had at least one infected fish based on simulations of PMCV spread in the Irish farmed Atlantic salmon population during 1 January 2009 to 23 October 2017. Time is represented in color [red [733 days] through to blue [1,060 days]]. The contours represent the time taken to reach 50% prevalence for a list of combinations of the x and y parameters. So, for example, a single line represents 900 days to reach 50% prevalence, which can be achieved by having the values of x and y shown in the plot. Parameter estimates were scaled from 0.5 to 1.5, by 0.1, including the indirect transmission rate parameters (vj,vs,vg, i.e., upsilon), decay of environmental infectious pressure (βq1, βq2, βq3, βq4, i.e., beta), the spatial coupling parameter (D, i.e., coupling), and the rate of viral shedding from infected individuals (α, i.e., alpha). The figure presents 6 different combinations of scaled parameters (e.g., the top left presents the impact on mean time to reach a between-farm prevalence of 50% or higher given different scaling factors for the decay of environmental infectious pressure (beta; on the x-axis) and the indirect transmission rate parameters (upsilon; on the y-axis). For each parameter combinations, the mean time to reach a between-farm PMCV prevalence of 50% or higher was calculated from 40 stochastic simulations. [file Image_1.JPEG]

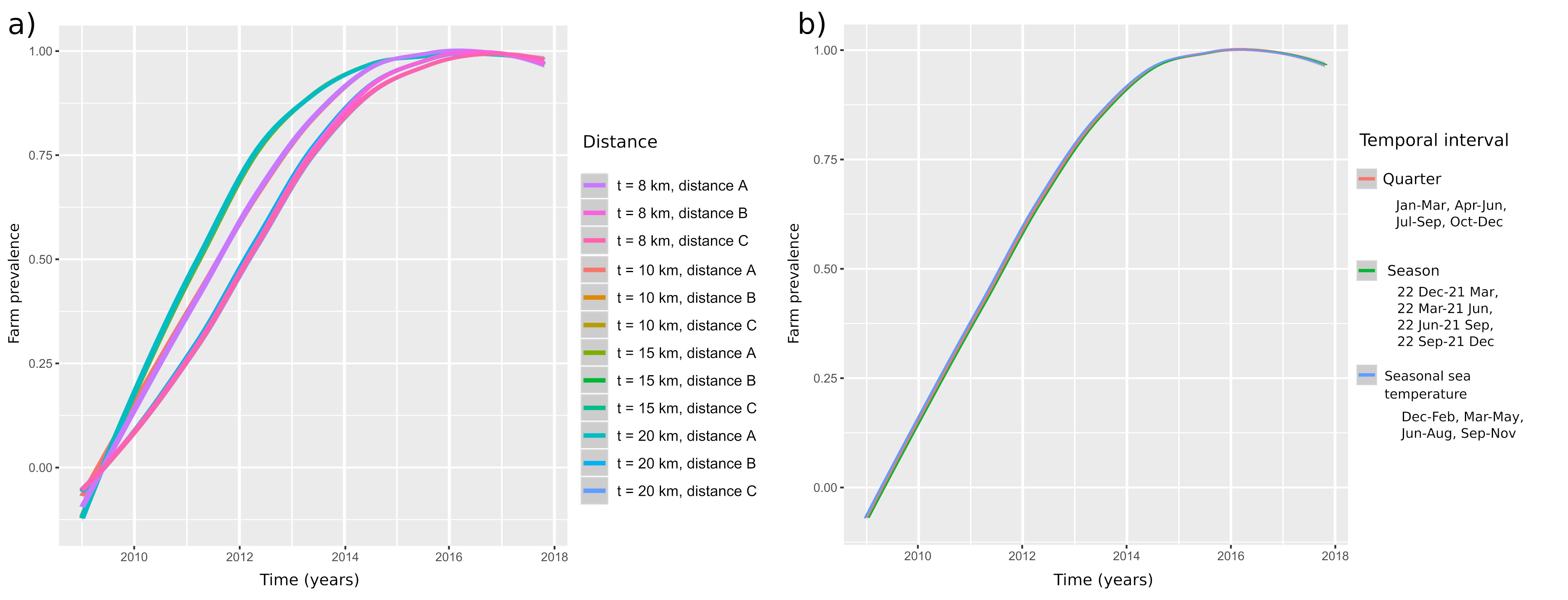

Supplement: Image S2 — Sensitivity analysis of the evolution of mean farm PMCV prevalence over time given different inputs for distance (a), left, and seasonality (b), right. The distance inputs included distance dependence [euclidean distance [A], 1/distance2 [B] and 1/distance3 [C]] and distance threshold value (8, 10, 15, 20 km). Therefore, “t = 8 km, distance A” refers to the use of euclidean distance during modeling with a distance threshold of 8 km, and “t = 20 km, distance B” refers to the use of 1/distance2 during modeling with a distance threshold of 20 km. The seasonality inputs included quarter (Jan-Mar, Apr-Jun, Jul-Sep, Oct-Dec), season (22 Dec-21 Mar, 22 Mar-21 Jun, 22 Jun-21 Sep, 22 Sep-21 Dec), and seasonal sea temperature (Dec-Feb, Mar-May, Jun-Aug, Sep-Nov). The plotted values correspond to the mean farm PMCV prevalence during 1 January 2009 to 23 October 2017, based on 40 stochastic simulations. [file Image_2.JPEG]
